# Supplementary material for: Content-rich biological network constructed by mining PubMed abstracts
Source: BMC Bioinformatics. 2004 Oct 8;5:147. doi: 10.1186/1471-2105-5-147 (PMC528731; doi:10.1186/1471-2105-5-147)
Supplement: Additional File 5 — The original Chilibot query results of the term "long-term potentiation (LTP)" and 22 other terms, limiting the latest references analyzed to the years 1990, 1995, 2000, and 2004. [file 1471-2105-5-147-S5.bz2 › chilibotAdditionalFile5/ltp2000/html/PLC_ACTININ.html]

 


 **PLC** and **ACTININ** 
  
Found 2 abstracts in PubMed,  **2 abstracts were retrieved and analyzed**.  


---

 Search Google  |
 PDF files only 
|  EDU domain only 

---

**Interactive relationship** (e.g. stimulation, inhibition, etc)

- Point mutants in which arginine 172 or lysine 184 of alpha  **actinin**  were replaced by isoleucine reduced the inhibitory effect on  **PLC**  activity by nearly half.  Ref: 8576235 J Biol Chem, 1996
- Furthermore, we detected an interaction between PIP2 and a bacterially expressed alpha  **actinin**  fragment amino acids 137 259 by  **PLC**  inhibition assay.  Ref: 8576235 J Biol Chem, 1996
